# Anaplastic Thyroid Carcinoma With Horner Syndrome and Unusual Metastasis Reveals Masked Papillary Thyroid Carcinoma Following Neoadjuvant Therapy

**DOI:** 10.1016/j.aed.2025.09.004

**Published:** 2025-10-21

**Authors:** Zain Akhtar, Humberto López Castillo, Deepa Taneja

**Affiliations:** 1 Burnett School of Biomedical Sciences, College of Medicine, Academic Health Sciences Center, University of Central Florida, Orlando, Florida; 2 Department of Health Sciences, College of Health Professions and Sciences, Academic Health Sciences Center, University of Central Florida, Orlando, Florida; 3 Department of Population Health Sciences, College of Medicine, Academic Health Sciences Center, University of Central Florida, Orlando, Florida; 4 Florida Diabetes, Thyroid, and Endocrine Center, Orlando, Florida

**Keywords:** anaplastic thyroid carcinoma, neoadjuvant therapy, papillary thyroid cancer, Horner syndrome, proto-oncogene proteins B-raf

## Abstract

**Background/Objective:**

Anaplastic thyroid carcinoma (ATC) is a rare thyroid cancer that has the propensity to be hyperactive and is characterized by the dedifferentiation of follicular cells leading to pleomorphism. This report highlights a 68-year-old man’s progression of ATC with an unusual presentation and secondary symptoms revealed to be masking an independent tumor cell origin residual papillary thyroid carcinoma following targeted treatment.

**Case Report:**

A 68-year-old man presenting with a history of aortic aneurysm and hyperlipidemia was referred for type 2 diabetes mellitus complications. He had a successful aneurysm repair; however, follow-up imaging revealed a thyroid mass, cystic lesions in the pancreas and liver, and extensive lymphadenopathy, and he endorsed symptoms of Horner syndrome. Fine-needle aspiration suggested ATC, and sequencing confirmed a *BRAF* V600E allelic variant. He began neoadjuvant *BRAF/MEK* inhibitor therapy to shrink the tumor and resolve metastasis. After a right thyroid lobectomy, pathology revealed a hidden non-*BRAF*-mutated papillary thyroid carcinoma. He is currently under multidisciplinary care and being considered for total thyroidectomy.

**Discussion:**

Normally, comorbid thyroid malignancies arise from identical follicular sequence variants. It is unusual to observe 2 independent origins from comorbid thyroid cancers. In addition, the development of Horner syndrome from thyroid cancer, especially anaplastic, is incredibly rare.

**Conclusion:**

This case is a unique depiction of how ATC can present despite uncharacteristic immunohistochemical markers, secondary symptoms, and hidden comorbidities. As an understudied malignancy, it is important to recognize this condition’s variability to prevent increased mortality and morbidity.

**Highlights**

• Papillary thyroid carcinoma is generally masked by anaplastic thyroid carcinoma (ATC)
• Papillary thyroid carcinoma can persist despite treatment if the allelic variant is different than ATC
• Horner syndrome is a rare complication of ATC prior to thyroidectomy
• Liver and pancreas metastasis is rarely seen in ATC

**Clinical Relevance**

This case reports a rare presentation of anaplastic thyroid carcinoma originating from a *BRAF* V600E sequence variant that was revealed to be masking a papillary thyroid carcinoma of independent tumor origin. This case emphasizes the need to identify each cancerous site’s origin for targeted treatment and identify causes of rare secondary symptoms.

## Introduction

Anaplastic thyroid carcinoma (ATC) is the rarest yet most fatal thyroid cancer, being responsible for nearly 50% thyroid cancer–related deaths.^1^ This hyperactive malignancy is characterized by pleomorphic and undifferentiated cells, metastasis, and lymph node positivity commonly in the cervical region.^1^^,^^2^ The most prevalent symptoms of ATC include dyspnea, dysphagia, and voice hoarseness arising from local invasion of neighboring structures, although clinical presentation remains variable.^2^ Horner syndrome (HS) is a neurologic disruption that normally results in unilateral ptosis, miosis, and anhidrosis. Although it develops similarly, HS is highly unlikely to develop from ATC.^3^

It has been proposed that ATC develops from a preexisting differentiated thyroid cancer or nodule; however, its progression following treatment has not been observed to revert to a lower stage of thyroid cancer, ultimately presenting with a coexisting thyroid malignancy.^1^^,^^4^ We report a case of a patient who underwent targeted therapy for ATC, ultimately unmasking a comorbid wild-type papillary thyroid carcinoma (PTC), along with a rare manifestation of HS.

## Case Report

A 68-year-old Hispanic man with past medical history of thoracic aortic aneurysm, chronic kidney disease, hyperlipidemia, and hypertension was referred for weight and type 2 diabetes management. Initial blood test results and measurements are found in Table 1. Outside of fatigue, he did not endorse symptoms indicative of a thyroid mass (eg, voice hoarseness, difficulty swallowing, or dyspnea), and his thyroid-stimulating hormone levels were unremarkable. After several months of using semaglutide 0.50 mg weekly, the patient’s parameters pertaining to management of diabetes and weight improved (Table 1), making him a suitable candidate for thoracic aortic aneurysm repair. Other prior medications included statins, antihypertensives, and loop diuretics.

**Table 1:** Laboratory Test Results

Laboratory test	7 mo preoperatively	2 mo preoperatively	4 mo s/p	Reference values
HbA1C (%)	6.6	6.0	6.8	<7% for well control
Fasting glucose (mg/dL)	159	96	83	65-99
BMI (kg/m^2^)	52.92	47.59	42.27	See note
TSH (mIU/L)	…	0.89	1.86	0.40-4.50
Free T_3_ (pg/mL)	…	3.7	2.9	2.3-4.2

Abbreviations: BMI = body mass index (a value of >40 signifies Class 3 obesity); HbA1C = glycated hemoglobin; s/p = status post thoracic aorta aneurysm repair; TSH = thyroid-stimulating hormone; T_3_ = triiodothyronine.

Three months after undergoing successful aortic aneurysm repair and being initiated on apixaban 5 mg twice daily, cardiology ordered a routine postoperative computed tomography angiography (CTA), which incidentally revealed an rapidly enlarging mass from the inferior aspect of the right thyroid lobe measuring 5.6 × 9.4 cm (previously 3.5 × 2.2 cm), a new 2.1-cm hypodense mass in the left hepatic lobe suggestive of hepatic metastasis, and a 2.9 × 1.4–cm cystic lesion in the pancreatic head (previously 2.2 cm). Subsequent imaging confirmed increased progression of metastases (Fig. *A* through *D*).

**Fig:**
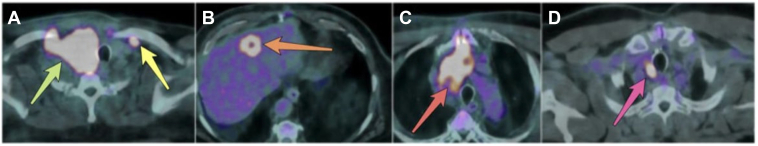
*A*, Positron emission tomography (PET)/computed tomography (CT) scan 2 weeks following fine-needle aspiration (FNA) diagnosis, confirming hypermetabolic activity in right thyroid (green arrow) as well as left supraclavicular lymph node (yellow arrow). The trachea was displaced due to enlarged thyroid mass. *B*, PET/CT scan 2 weeks following FNA diagnosis showing intense fluorodeoxyglucose (FDG)-avid lesion along with central necrosis located on the left side of the liver (orange arrow). *C*, PET/CT scan 2 weeks following FNA diagnosis, emphasizing increased FDG uptake causing mediastinal para-aortic lymphadenopathy along with thyroid mass extending inferiorly into the mediastinum (red arrow). *D*, PET/CT scan 1 month status post right thyroid lobectomy, emphasizing increased FDG uptake in right paratracheal lymph node (pink arrow) stemming from residual papillary thyroid carcinoma.

A fine-needle aspiration biopsy of the thyroid mass was performed after the CTA, initially suggesting poorly differentiated thyroid carcinoma (PDTC). Updated immunohistochemistry (Table 2) along with pleomorphic epithelioid cells and significant nuclear atypia confirmed ATC with a pathologic T4aN1bM1 stage IVC. Next-generation sequencing (NGS) was immediately started, identifying the *BRAF* V600E sequence variant (variant allele fraction of 0.532).

**Table 2:** Immunohistochemical Results From Fine-Needle Aspiration of Right Thyroid Mass

Immunohistochemical markers	Expression
MOC-31	+
CK5/6	++
p40	Negative
PAX8	+
Thyroglobulin	Negative
p53	+++
SOX10	Negative
Smooth muscle actin	Negative
S-100	Negative
MIB-1 - Ki-67 proliferation	Approximately 25%
TTF-1	+

+, immunopositivity; ++, focal weak to moderate immunopositivity; and +++, diffuse overexpression.

In the interim, the patient endorsed symptoms of hemoptysis, ptosis, miosis, and right-sided facial anhidrosis, the latter 3 consistent with HS. A magnetic resonance imaging scan of the brain was ordered and unremarkable, thus ruling out possible brain metastasis or oculomotor or trigeminal nerve impairment. Physical examination showed a noticeable cervical mass, and positron emission tomography scans revealed aggressive cancer progression causing bilateral cervical, supraclavicular, and mediastinal lymphadenopathy (Fig. *B*), alongside hypermetabolic bilateral pulmonary nodules consistent with ATC metastasis.

Following confirmation of the *BRAF* V600E variant, the patient discontinued both apixaban and semaglutide and was initiated on dabrafenib 150 mg twice daily and trametinib 2 mg daily. The patient’s hemoptysis and HS symptoms stopped within weeks. In addition to affected lymph nodes, areas with ATC metastasis (ie, liver, pancreas, and lung) had noticeably reduced metabolic activity, and the thyroid mass shrank to 2.3 × 5.3 cm, allowing for surgical resection. The patient’s appearance and physical examination noticeably improved during the targeted treatment.

The patient consented to a right thyroid lobectomy nearly 7 months after beginning neoadjuvant therapy. The final pathology report of the resected thyroid lobe revealed a residual tall cell variant PTC. Further positron emission tomography scans (Fig. *C*) revealed lymphadenopathy in the right paratracheal lymph node and level II lymph node. The residual PTC did not test positive for *BRAF* mutation burden in the NGS panel.

The patient’s thyroid markers have remained stable without supplementation but show diminished follicular cell characteristics (Table 3). The patient is currently involved in multidisciplinary discussion among his care team, considering a total thyroidectomy due to residual cancer.

**Table 3:** Thyroid Markers Following Lobectomy

Laboratory test	1 wk s/p	3 mo s/p	Reference values
TSH (mIU/L)	2.08	2.14	0.40-4.50
Free T_3_^(pg/mL)^	3.2	3.1	2.3-4.2
Thyroglobulin (ng/mL)	0.5	1.0	3-40
TgAb (IU/mL)	210	250	≤1

Abbreviations: s/p ^=^ status post right thyroid lobectomy; TgAb = thyroglobulin antibodies; TSH = thyroid-stimulating hormone; T_3_ = triiodothyronine.

## Discussion

Although the process of ATC forming from a preexisting thyroid nodule or other follicular cell thyroid cancer has been recognized in the literature, we present a case where the successful targeted treatment of ATC led to unmasking a hidden comorbid PTC of independent tumor cell origin. Historically, the preexisting ATC following treatment is either resolved, unresolved leading to fatal outcomes, or comorbid with other thyroid cancers, such as PTC or PDTC.^5^^,^^6^ Despite the appearance of the residual PTC, the patient did not have any prior history of thyroid cancer, had only 1 suspicious nodule at time of ATC diagnosis, and was not observed to have multiple unique (differentiated and undifferentiated) thyroid malignancies at once prior to surgical resection. No thyroid imaging (eg, ultrasound or scans) was performed prior to postoperative CTA.

The patient using semaglutide (a glucagon-like peptide-1 receptor agonist) 10 months prior to thyroid cancer diagnosis poses a theoretical risk in developing a thyroid malignancy, as seen in animal studies. However, human data have been inconclusive in correlating to a higher incidence of any type of thyroid cancer.^7^^,^^8^

The immunohistochemistry of the patient’s fine-needle aspiration, as seen in Table 2, confirms immunopositivity of PAX8, diffuse p53 expression, and a relatively high Ki-67 proliferation index. This, along with thyroglobulin absence, supported the diagnosis of ATC.^6^ Although the immunopositivity of TTF-1 is atypical in ATC, it may persist if its positivity is present focally, the tissue displays prominent pleomorphism, and squamoid components of epithelioid cells are present, all of which occurred in the following case, thus ruling out the presence of PDTC in the patient.^6^^,^^9^

*BRAF* V600E sequence variations are very commonly observed in ATC.^10^ With it being the only positive variant identified, combination therapy of dabrafenib (*BRAF* inhibitor) and trametinib (inhibitor of mitogen-activated protein kinase or *MEK*) was used. This treatment has been shown to increase the median overall survival from a historically reported <6 months to approximately 14.5 months.^11^ The median overall survival is noted to be approximately 3 months for those diagnosed with stage IVC ATC.^12^ The patient’s first dose of *BRAF/MEK* inhibitors was recorded to be 10 months after his first visit, expected to be approaching over a year. Analysis of the residual PTC revealed that it did not test positive for the *BRAF* V600E allelic variant, explaining why the targeted therapeutic approach was ineffective toward it. Residual PTCs are generally excised because of total thyroidectomies being performed; however, there is very little literature discussing the significance of dealing with the cancer after a lobectomy.^13^ There is discussion of the patient undergoing radiation therapy due to proximal metastasis and lymphadenopathy due to the PTC.

The presence of HS in a preoperative setting is a rare complication of thyroid etiology with scant literature addressing the matter.^3^ There is much more discussion on HS development postoperatively, related to thyroid cancers in cases where either a neck dissection, thyroidectomy, or cervical lymphadenectomy is required. It is estimated the 1.3% of cases of HS stem from thyroid pathology, which does not exclude postoperative complications.^14^^,^^15^ ATC is expected to be the cause of <0.1% of all HS cases in a preoperative scenario. The patient’s enlarged thyroid mass (maximum dimensions at 5.6 × 9.4 cm) led to preganglionic neuron damage to his right oculosympathetic nerve pathway. It is recommended to treat HS by addressing the underlying cause first, and the patient’s HS was resolved after the reduction in size of his thyroid mass following *BRAF/MEK* inhibitor therapy. HS can be misdiagnosed with cranial nerve impairment (either oculomotor or trigeminal) and was ruled out following patient’s brain magnetic resonance imaging.^16^

Approximately 50% of ATC cases present with metastasis at diagnosis.^17^ The patient presented with common sites of metastasis in his lungs (78%) and cervical lymph nodes (51%) but also observed 2 other infrequent sites of metastatic progression in his liver (Fig. *D*) featuring (20%) and pancreas (4%).^18^ These 2 sites were concerning due to the patient’s history of comorbid diabetes and hyperlipidemia. All metastatic sites were resolved shortly after *BRAF/MEK* inhibitor treatment, and the patient has resumed his statin usage while adding empagliflozin/metformin to his regimen for further diabetic management.

ATC manifests and presents in a multitude of ways that should be clinically recognized, with increasing number of possibilities in malignancy progression. This case report highlights the complexity of diagnosing comorbid thyroid malignancies in a patient whose ATC was incidentally diagnosed with a routine CTA, ultimately masking the presence of any underlying cancer. NGS panel should be initiated early on in diagnosis of thyroid cancer, potentially salvaging the development of the cancer to rare metastatic sites depending on mutation burden. Although rare, clinicians should be aware of conditions caused by ATC tumor compression to cervical neuronal pathways such as HS. See Supplementary Figure “Visual Timeline.”

## Conclusion

ATC presents with various unexpected modalities and can form due to aggressive dedifferentiation of follicular cells; however, coexisting thyroid malignancies from independent origins are rare and often indistinguishable. This case highlights the significance of early NGS testing and coordinated multidisciplinary care to detect masked malignancies and guide targeted therapy. Such approaches can help clinicians tailor treatment for aggressive thyroid cancers while providing researchers with data to refine diagnostics and optimize therapeutic strategies for better patient outcomes.

## Disclosure

The authors have no conflicts of interest to disclose.
